# TRIP/TWIP Networks Promoted via Multifunctional Nanoprecipitates‐confined Specified Shear for Achieving Strong‐yet‐Ductile Titanium Alloys

**DOI:** 10.1002/advs.202511834

**Published:** 2025-09-22

**Authors:** Xiaofu Zhang, Shu Wang, Ruirun Chen, Minghao Hua, Weipeng Xu, Hongwei Wang, Shuo Yin

**Affiliations:** ^1^ National Key Laboratory for Precision Hot Processing of Metals School of Materials Science and Engineering Harbin Institute of Technology Harbin 150001 P. R. China; ^2^ School of Energy and Power Engineering Shandong University Jinan 250061 P. R. China; ^3^ Department of Mechanical Manufacturing and Biomedical Engineering Trinity College Dublin The University of Dublin Dublin 2 Ireland

**Keywords:** density functional theory, Lüders deformation, titanium alloys, TRIP/TWIP effects, ω‐nanoprecipitates

## Abstract

Transformation‐induced plasticity (TRIP) and twinning‐induced plasticity (TWIP) are typically suppressed by precipitates and difficult to be significantly triggered under high yield stress. In titanium alloys, ω phase with intrinsic deformation heterogeneity localizes deformation to {112}〈111〉_β_ system, exactly aligning with the lattice shear in martensite transformation (MT). Therefore, beyond reported ω‐nanoprecipitates functions, e.g., providing precipitation strengthening, maintaining strain compatibility and dynamically forming ω‐free dislocation channels, new confining specific shear is proposed to integrate precipitates with TRIP/TWIP effects in this work. A de novo design scheme, consisting of Density Functional Theory, Cluster Expansion, Monte Carlo simulations and Ab Initio Molecular Dynamics, is employed for composition screening. β phase stability and β‐ω continuous slip barriers are precisely tailored to provide large chemical driving force for MT while suppressing excessively slip priority. After simple thermomechanical processing, selected Ti‐7.92Mo‐3.22Cr‐1.88Zr alloy exhibits dense TRIP/TWIP networks and record yield strength‐ductility synergy (product exceeding 38 GPa%). Premature necking is delayed by ω‐confined elevated local stress promoting MT followed by sequential transformation from strain‐induced martensite to {332}〈113〉_β_ deformation twins, thus forming an extended ≈23.2% Lüders‐type strain. These theoretical and experimental results provide implementable and individual strategies to overcome yield strength‐ductility trade‐off by reconciling precipitation strengthening with TRIP/TWIP effects.

## Graphical abstract

Comprehensive theoretical calculation methods are employed for composition design to manipulate precipitates with deformation heterogeneity for facilitating TRIP/TWIP effects. Selected alloy exhibits dense TRIP/TWIP networks at > 830 MPa yield stress and record yield strength‐ductility synergy in titanium alloys. The results offer an individual pathway to break the low yield strength cruse of TRIP/TWIP‐type alloys without sacrificing ductility.

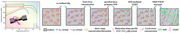

## 1 Introduction

High yield strength (but restricted ductility) and large ductility (yet low yield strength) can be achieved by obstructing or facilitating dislocation activity in metallic structural materials.^[^ ^1^ ^]^ However, even a well‐designed resistance to dislocation activities is still confined to sliding within the banana‐shaped yield strength‐ductility loop, thereby rendering the synergistic enhancement of yield strength and ductility unattainable.^[^ ^2^ ^]^ It can be naturally estimated that at high yield strength, the work hardening supplied solely by a restricted dislocation activity is insufficient to prevent the premature initiation of necking under uniaxial tension.^[^ ^3^ ^]^

Metastability engineering, encompassing transformation‐induced plasticity (TRIP) and twinning‐induced plasticity (TWIP), which alleviates stress concentrations and induces the dynamic Hall‐Petch effect to provide strong work‐hardening contributions, is an effective strategy for enhancing ductility.^[^ ^4^ ^]^ The TRIP/TWIP effects are predominantly introduced by the single metastable β phase in titanium alloys,^[^ ^5^ ^]^ whereby ductility can exceed 50% even at unsatisfactory yield strength.^[^ ^6^ ^]^ Since the 2010s, TRIP/TWIP‐Ti alloys have progressively evolved into a promising new family of titanium alloys,^[^ ^7^, ^8^ ^]^ with extensions demonstrated in Zr‐based alloys.^[^ ^9^, ^10^ ^]^ The enhancement of the yield strength through fine‐grain strengthening in TRIP/TWIP‐Ti alloys is considered suboptimal and complex, because of the nonlinear relationship between the grain size and the triggering stress of the TRIP/TWIP effects.^[^ ^11^, ^12^ ^]^ Moreover, other strengthening approaches that effectively impedes dislocation activities are commonly accompanied by an increase in the triggering stress of TRIP/TWIP effects, such as high solid‐solution atomic concentrations,^[^ ^13^, ^14^ ^]^ significant secondary phases,^[^ ^15^, ^16^ ^]^ and cold deformation.^[^ ^17^ ^]^ Therefore, activating abundant TRIP/TWIP effects at high yield strength remains challenging for the development of TRIP/TWIP‐Ti alloys.

The ingenious integration of heterogeneity design and metastability engineering provides the new opportunities for obtaining strong yet ductile in titanium alloys. The alternating activation of strain‐induced martensite (SIM) and deformation twins (DT) can be induced by heterogeneous solute distribution within the grain interiors.^[^ ^18^ ^]^ The heterogeneous grain size and dislocation density could synergistically improve the yield strength and ductility by changing the triggering sequence of dislocation slip and DT.^[^ ^19^ ^]^ The advantages of heterogeneity design and metastability engineering can be fully leveraged using multifunctional nanoprecipitates. Zhang et al.^[^ ^20^ ^]^ reported that a heterogeneous laminated structure, which was outlined by nanoscale α precipitates, doubled the yield strength with only a minor compromise in plasticity, as compared with the coarse‐grained state. The nanoprecipitates reportedly possess three functionalities, namely, activation of the TRIP effect in the soft matrix, precipitation strengthening and interfacial delamination toughening.

Considering the preparation complexity of above heterostructures (solute distribution, grain size, dislocation density and phase distribution) as well as the strain incompatibility at semi‐coherent α/β interfaces,^[^ ^21^ ^]^ we redirect attention toward coherent ω phase with intrinsic deformation heterogeneity. Well‐distributed coherent ω‐nanoprecipitates can be readily obtained in metastable β‐Ti alloys through a solution treatment followed by water quenching, referred to as a thermal ω (ω_ath_) phase.^[^ ^22^ ^]^ Conducting by low‐temperature and short‐time aging (LTSTA), the ω_ath_ transforms into the early stage of isothermal ω (ω_iso_) phase, accompanied solely by solute short‐range diffusion.^[^ ^16^, ^23^ ^]^ In this state, the ω phase not only provides precipitation strengthening but also maintains the stability of the matrix at significant distances from the β/ω interfaces.^[^ ^16^, ^24^ ^]^ More importantly, the formation of SIM at plastic stage is closely correlated with the dislocation activity in the same slip system.^[^ ^25^, ^26^ ^]^ The intrinsic deformation heterogeneity of the ω phase confines β phase slip exclusively to the {112}<111> system,^[^ ^24^, ^27^, ^28^, ^29^, ^30^ ^]^ which precisely coincides with the shear direction of martensite transformation (MT; β→α′/α′′).^[^ ^11^, ^31^ ^]^ That is, high local stress from ω‐induced localized deformation amplifies the possibility of triggering MT. Furthermore, the ω‐free dislocation channels formed owing to dislocation pile‐ups are expected to promote dislocation strengthening and the TRIP/TWIP‐induced dynamic Hall‐Petch effects to yield sufficient work‐hardening capacity.^[^ ^24^, ^27^, ^28^, ^29^, ^30^ ^]^

This study used the Ti‐Mo‐Cr‐Zr system was selected as a paradigm to demonstrate a de novo design concept and the plastic deformation behavior of the multifunctional ω phase (providing precipitation strengthening; maintaining strain compatibility; dynamically forming dislocation channels; confining specified shear) enhanced TRIP/TWIP‐Ti alloys. The β phase stability and unstable stacking fault energies along the β‐ω continuous slip system were carefully modulated to prioritize TRIP/TWIP effects over dislocation slip. The addition of Cr (stronger β‐stabilizing effect than Mo) and Zr (weaker stabilizing effect than Mo) with different atomic radius helped to readily achieve an ideal combination of β phase stability and β‐ω continuous slip barriers. The LTSTA‐processed specimen, as compared to the coarse‐grained solution‐treated specimen, exhibited ≈80% increased yield strength yet enhanced TRIP/TWIP activity, thereby achieving a record yield strength‐ductility synergy. We emphasize accomplishing the synergistic integration between heterogeneous deformation precipitates and TRIP/TWIP effects via composition design. This approach offers a novel strategy to overcome the strength‐ductility trade‐off in metallic structural materials.

## 2 Results and Discussion

### 2.1 De Novo Composition Design Scheme

The target properties to be addressed by the composition design were first identified. After solution treatment with subsequent water quenching, the well‐dispersed ω phase exhibits the size of 3–5 nm in metastable β‐titanium alloys with various compositions.^[^ ^32^ ^]^ Furthermore, there is no discernible alterations except for solute short‐range diffusion in the microstructures characteristics of ω phase following LTSTA treatments.^[^ ^16^, ^33^ ^]^ Such simple and stable initial microstructures are demonstrated to highlight the dominance of composition‐determined physical properties in ω‐enhanced TRIP/TWIP‐Ti alloys. In particular, the response of the ω phase to LTSTA treatments and the potential for activating TRIP/TWIP effects are dominated by the β phase stability during ω‐transition and martensite transformation (MT), respectively.^[^ ^32^, ^34^ ^]^ The lattice shear during MT can be triggered by the elevated local stress originating from ω‐confined localized deformation, which is dependent on the β‐ω continuous slip barriers.^[^ ^28^, ^35^ ^]^ The precipitation strengthening effect of ω phase is mainly attributed to modulus hardening and stacking fault strengthening, as compared to lower coherency strengthening.^[^ ^36^, ^37^ ^]^

**Figure 1:**
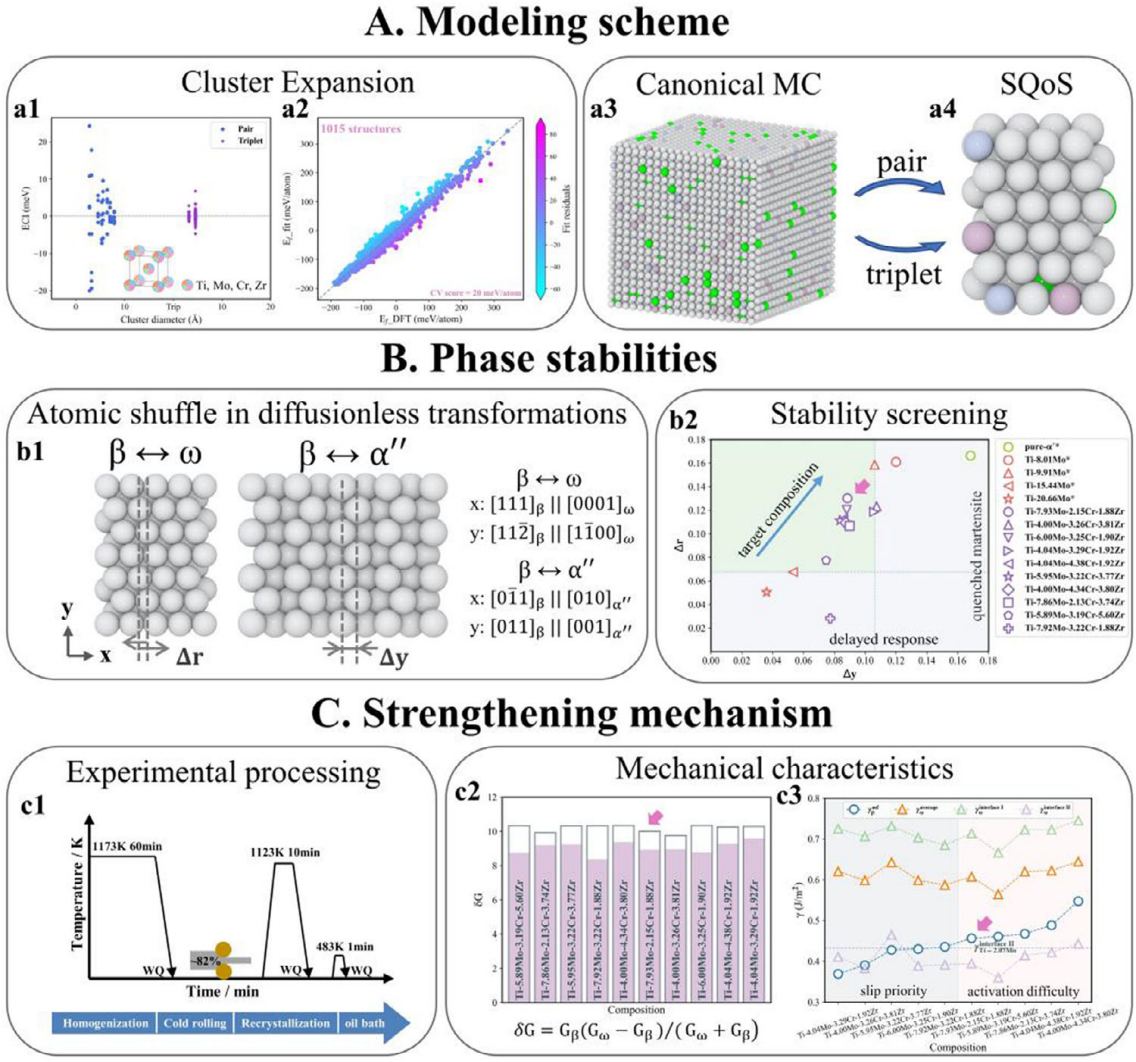
De novo composition design scheme. a1) the effective cluster interactions variation as a function of the clusters interaction distance in Ti‐Mo‐Cr‐Zr system. Inset shows a schematic diagram illustrating the lattice points occupation in CE calculations. a2) the comparison of energies from the CE predictions and DFT calculations for the same configuration in Ti‐Mo‐Cr‐Zr system. a3,a4) large‐scale canonical ensemble MC simulation snapshot and small‐scale SQoS configuration. b1) schematic diagram of atomic shuffle during ω‐transition (Δr) and MT (Δy). b2) combination of Δr and Δy values to screen the alloy compositions with large chemical driving force. c1) schematic diagram illustrating the alloy processing parameters. c2) shear modulus mismatch between β and ω phases under different compositions. c3) the unstable stacking fault energies of β (the average of 12 planes in supercell, $\gamma_{\beta }^{\mathrm{usf}}$), ω_1_ (the average of 12 planes in supercell, $\gamma_{\omega }^{\mathrm{average}}$), non‐continuous slip planes in ω_1_ (the average of 8 planes, $\gamma_{\omega }^{\mathrm{interface}\;I}$) and continuous slip planes in ω_1_ (the average of 4 planes, $\gamma_{\omega }^{\mathrm{interface}\;II}$). The data in b2 marked with asterisks was derived from [our unpublished results].

Based on the targeted thermodynamic and kinetic properties of phases proposed above, a comprehensive computational scheme was employed for composition screening. **Figure** **1**a1–a4 illustrate the procedure for constructing density functional theory (DFT)‐scale representative structures incorporating solute‐atom distribution features. In particular, cluster expansion (CE) and Monte Carlo (MC) simulations were performed to obtain large‐scale thermodynamically equilibrium configurations at the experimental homogenization temperature, from which pair and triplet clusters correlations were extracted as the objective function to generate DFT‐scale representative structures via special quasiordered structure (SQoS) method. The modeling scheme aimed to precisely reproduce the experimental atomic environment following the homogenization treatment as closely as possible.

As previously mentioned, only solute short‐range diffusion occurs during LTSTA treatments, therefore, the phase stability of the β‐matrix away from β/ω interfaces can be simply assessed using nominal compositions as the simplified approach. Figure 1 (b1, b2) show the evaluation method used to assess the β phase stability during two diffusionless transformations. Given the dynamical/mechanical instabilities of β‐Ti at 0 K,^[^ ^38^ ^]^ crystal structure transition degree was employed to indirectly infer the thermodynamic stability of β phase.^[^ ^39^, ^40^ ^]^ As shown in Figure 1(b1), the Δr and Δy were defined as the average atomic shuffle during ω‐transition and MT, respectively. The spontaneous lattice strain (SLS) represented the degree of lattice shear during MT.^[^ ^41^ ^]^ The Δr and Δy were jointly selected for phase stability screening due to the near‐linear relationship between Δy and SLS (Figure S1, Supporting Information, the deviation of the linear relationship from the origin implies the existence of O' nanodomains^[^ ^31^ ^]^). Moreover, the pure‐Ti and Ti‐Mo binary systems were used as reference standards [our unpublished results]. The Δr values (Figure 1 b2) must exceed that of Ti‐15.44Mo (wt.%, and same hereafter) to avoid ω‐embrittlement risks arising from sluggish response to LTSTA treatment.^[^ ^33^, ^42^ ^]^ Meanwhile, the Δy values (Figure 1 b2) must be lower than that of Ti‐9.91Mo (the concentration of the current supercell models cannot be strictly taken for Ti‐10Mo) to prevent the formation of quenched martensite.^[^ ^43^ ^]^ Based on pre‐screening using conventional phenomenological methods,^[^ ^34^ ^]^ the Δy values of most compositions locate the region between Ti‐9.91Mo and Ti‐15.44Mo, where TRIP/TRIP effects are most abundantly triggered.^[^ ^43^, ^44^ ^]^ In summary, the compositions proximate to the upper‐right corner of the green‐shaded region in Figure 1 (b2) were identified as the target compositions that exhibit significant chemical driving forces for both diffusionless phase transformations.

The assessment of the precipitation strengthening effects cannot neglect the composition variation from solute short‐range diffusion in nanoscale ω phase, which depends on temperature and duration of LTSTA treatments.^[^ ^33^, ^45^ ^]^ Following short‐range diffusion, the solutes concentration wave formed from the ω‐core region to the β/ω interface.^[^ ^45^, ^46^ ^]^ The solute‐rich regions in the ω phase were simply assumed to maintain the nominal composition of alloys, while solute‐lean regions reach the partitioning limit. The solute partitioning limit in Ti‐12Mo alloy was identified as Ti‐1.38 at.% Mo.^[^ ^33^ ^]^ In current work, it was set as Ti‐1.04 at.% Mo (equivalent to Ti‐2.07 wt.% Mo, represented by one Mo‐atom doped in the 96‐atom supercell). Furthermore, according to experimental results of LTSTA‐treated (473 K / 60 s) Ti‐12Mo alloy,^[^ ^16^ ^]^ volume fractions of Ti‐rich clusters (*c*_Mo_ = 3.62 at.%, refer to ω phase) and Mo‐rich clusters (*c*_Mo_ = 39.91 at.%, refer to β‐matrix) were identified as 4% and 12%, respectively. This indicates that ≈84% of the alloy remains near nominal composition (if any Mo‐rich regions exceeding nominal composition but below 39.91 at.% persist in the matrix, the alloy composition would overshoot nominal composition). Consequently, we assigned nominal composition to β‐matrix bulk and ω‐core during calculations.

Considering the lower Δr values and potential multi‐component diffusion retardation effects in the Ti‐Mo‐Cr‐Zr system, as compared with Ti‐12Mo alloy, the temperature of LTSTA treatments increased by 10 K relative to the optimal temperature for Ti‐12Mo alloy,^[^ ^16^ ^]^ as shown in Figure 1 (c1). More details on LTSTA process parameters selection and its potential implications for thicker sections were provided in Notes S1, S2 and Figures S2 and S3 (Supporting Information). In metastable β‐Ti alloys, lattice shear strain of SIM was governed by the lattice stability of β parent phase.^[^ ^47^, ^48^ ^]^ However, the Young's modulus and shear modulus of ω phase are more than double that of β phase (Figure S4, Supporting Information), which significantly enhances the lattice stability of β parent phase.^[^ ^24^ ^]^ Therefore, δG was defined to mainly evaluate the strengthening effect caused by shear modulus mismatch (Figure 1c2), according to the force between dislocations and precipitates.^[^ ^37^, ^49^ ^]^ For β‐matrix and ω phase with solute partitioning limits, δG values exhibit similarity across compositions, as indicated by the total bar heights in Figure 1 (c2). Notably, Ti‐7.92Mo‐3.22Cr‐1.88Zr exhibits a significantly lower δG under non‐partitioned conditions, as reflected by the filled bar heights in Figure 1 (c2). This is attributed to the structural similarity between β and ω phase resulting from high β phase stability of Ti‐7.92Mo‐3.22Cr‐1.88Zr during ω‐transition, as shown in Figure 1 (b2).

Consistent with previous studies,^[^ ^28^, ^35^ ^]^ the unstable stacking fault energies (USFEs) were calculated to evaluate the energy barriers for β‐ω continuous slip along $\left(1\bar{1}2\right){\left[\bar{1}11\right]}_{\beta }$ and $\left(\bar{2}020\right){\left[0001\right]}_{\omega }$, which is defined as $\gamma_{\beta }^{\mathrm{usf}}$ and $\gamma_{\omega }^{\mathrm{interface}\;II}$ (non‐partitioned conditions; $\gamma_{\mathrm{Ti}-2.07\mathrm{Mo}}^{\mathrm{interface}\;II}$ for partitioning limit conditions) respectively (Figures S5 and S6, Supporting Information). The significantly higher USFEs of non‐continuous slip planes in ω phase (defined as $\gamma_{\omega }^{\mathrm{interface}\;I}$), as compared with $\gamma_{\omega }^{\mathrm{interface}\;II}$ and $\gamma_{\beta }^{\mathrm{usf}}$ demonstrate its intrinsic deformation heterogeneity. Additionally, we also calculated regarding solute partitioning limit for interfacial β‐matrix,^[^ ^16^ ^]^ as shown in Table S1 (Supporting Information). Expectedly, it also contributed to alloy strength due to higher USFEs and shear modulus. However, both triggering of SIM and activation of slip should be primarily governed by the β‐matrix bulk. Therefore, particular focus was placed on beta‐matrix bulk and ω phase. The influences of USFEs presents a duality, similar to the dilemma of dislocations motion in the strength‐ductility trade‐off: on one hand, the occurrence of continuous slip is facilitated by low $\gamma_{\beta }^{\mathrm{usf}}$ and $\gamma_{\omega }^{\mathrm{interface}\;II}$, further forming more ω‐free dislocation channels, as indicated by the gray fill region in Figure 1 (c3); conversely, large USFEs mismatch (light pink region in Figure 1 c3) is beneficial for enhancing strength by increasing the trigger stress of dislocation slip.^[^ ^37^ ^]^ Notably, in the target region within Figure 1 (b2), the TWIP effect (specifically predominant {332}〈113〉_β_ deformation twins in TRIP/TWIP Ti alloys,^[^ ^50^ ^]^ that is, 332DT) is anticipated to be mediated by SIM rather than being triggered directly.^[^ ^51^ ^]^ Therefore, activating abundant TRIP/TWIP effects requires ensuring the occurrence of slip to provide an elevated local stress for MT‐essential shear while suppressing its excessive precedence. Both extremely high $\gamma_{\beta }^{\mathrm{usf}}$ and low continuous slip energy barriers are undesirable. Accordingly, Ti‐7.93Mo‐2.15Cr‐1.88Zr was selected as the optimal composition (pink arrow in Figure 1 c3) that simultaneously balances a minimized β phase stability to enable large chemical driving force in two diffusionless transformations (pink arrow in Figure 1 b2) and an effective modulus mismatch to achieve satisfactory precipitation strengthening (pink arrow in Figure 1 c2).

### 2.2 Microstructure and Mechanical Properties

**Figure 2:**
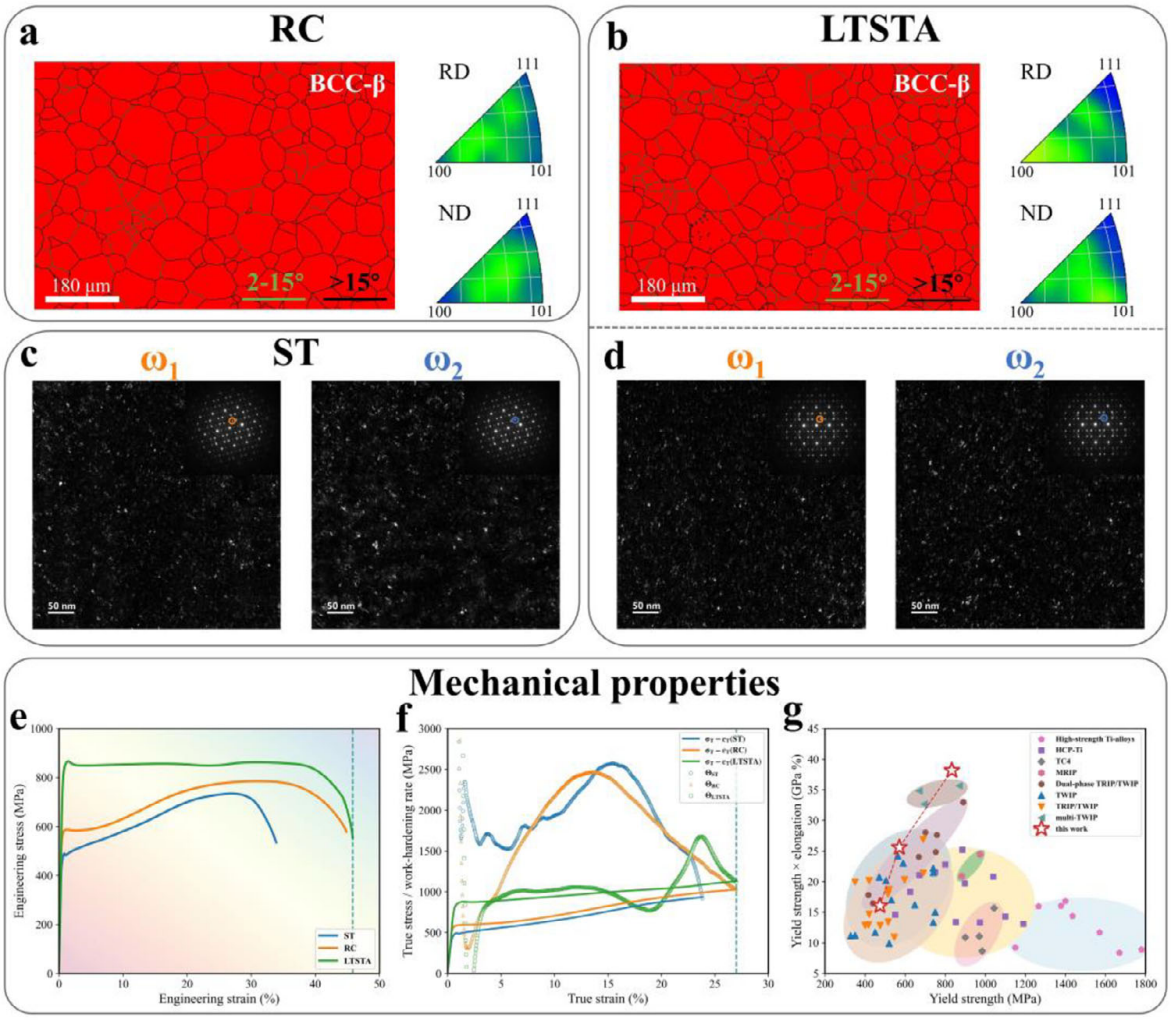
Initial microstructures and mechanical properties of Ti‐7.93Mo‐2.15Cr‐1.88Zr alloy under various conditions. a,b) phase maps and inverse pole maps of the initial microstructures of the RC and LTSTA specimens, respectively. c,d) dark field images of two ω‐variants in initial microstructures for ST and LTSTA specimens. Insets exhibit the corresponding selected area electron diffraction (SAED) patterns. e,f) engineering stress‐strain, true stress–strain, and work‐hardening rate curves for ST, RC, and LTSTA specimens. g) yield strength‐ductility comparison of Ti‐7.93Mo‐2.15Cr‐1.88Zr alloy with other titanium alloys. Data extracted from engineering stress‐strain curves of reported typical titanium alloys, that is, high‐strength alloys,^[^ ^52^, ^53^, ^54^, ^55^, ^56^, ^57^ ^]^ HCP‐Ti,^[^ ^58^, ^59^, ^60^, ^61^ ^]^ TC4,^[^ ^62^, ^63^, ^64^, ^65^ ^]^ MRIP‐Ti alloys,^[^ ^66^ ^]^ dual‐phase TRIP/TWIP‐Ti alloys,^[^ ^20^, ^67^, ^68^, ^69^ ^]^ TWIP‐only Ti alloys,^[^ ^70^, ^71^, ^72^, ^73^, ^74^, ^75^, ^76^, ^77^, ^78^ ^]^ TRIP/TWIP‐Ti alloys,^[^ ^24^, ^74^, ^77^, ^79^, ^80^, ^81^, ^82^, ^83^, ^84^, ^85^, ^86^ ^]^ and multi‐TWIP Ti alloys.^[^ ^87^, ^88^, ^89^ ^]^

Our target microstructure comprises β‐matrix with ω‐nanoprecipitates, where the grain size of the former and microstructure characteristics of the latter are key considerations for MT.^[^ ^11^, ^16^ ^]^ As depicted in Figure 1 (c1), Ti‐7.93Mo‐2.15Cr‐1.88Zr alloy underwent successive processing, namely, solution treatment (marked as ST specimen), cold rolling, recrystallization treatment (marked as the RC specimen), and low‐temperature and short‐time aging treatment (marked as the LTSTA specimen). **Figure** **2**a,b reveal no marked differences in either the grain size (RC specimen: 49.6 µm; LTSTA specimen: 49.0 µm) or crystallographic orientations of β‐matrix after LTSTA treatment. Obviously, temperature (483 K) far below β‐transus would not drive grain coalescence and coarsening for β‐matrix. In fact, 483 K corresponds to the ω_iso_ transformation stage,^[^ ^32^ ^]^ yet as noted above, such short duration (60s) permits only short‐range diffusion of β‐stabilizers from ω phase to β‐matrix. Figure 2c,d displayed two identifiable ω‐variants in undeformed ST and LTSTA specimens. Quantitative analysis^[^ ^28^, ^29^ ^]^ based on our transmission electron microscope (TEM) results showed no obvious differences in size, number density, volume fraction, or spacing of ω phase across the three states, as exhibited in Table S2 (Supporting Information). Furthermore, we selected Ti and Mo with higher concentrations for compositional analysis since β‐stabilizers would be expelled during ω_iso_ growth. As shown in Figure S7 (Supporting Information), no identifiable change occurred in fluctuation frequency of elemental concentration after LTSTA treatment, while the amplitude of that distinctly increased. Consequently, short‐range diffusion occurred between ω phase and β‐matrix without achieving long‐range equilibrium after LTSTA. These conclusions on microstructure characteristics and composition features aligned with reported literatures on LTSTA treatment,^[^ ^16^, ^23^ ^]^ i.e., short‐range concentration fluctuations between matrix and ω phase formed after LTSTA treatment would significantly enhance yield strength.

Figure 2e shows the engineering stress‐strain (σ_e_‐ε_e_) curves for three treated states, demonstrating synergistic enhancement in both yield strength (σ_y_, 0.2% proof engineering stress) and fracture elongation (ε_f_, measured using the engineering strain) from ST, RC to LTSTA states (σ_y_: 474.1→570.7→832.9 MPa; ε_f_: 33.9→44.9→45.8%). The LTSTA specimen exhibits typical Lüders‐type strain characterized by a ≈16.2 MPa yield drop with a ≈23.2% ε_e_ Lüders plateau. This is clearly manifested in work‐hardening rate (WHR) curves, as shown in Figure 2f. Within the Lüders plateau, WHR slightly exceeded true stress (σ_T_) with nearly parallel trajectories, preventing rapid necking until fracture. Following Lüders plateau, LTSTA specimen initiated uniform deformation with ≈1.7 GPa WHR peak. As shown in Figure S8 (Supporting Information), DIC testing reveals strain fronts propagating at ≈30°, consistent with SIM‐induced Lüders‐type strain.^[^ ^12^, ^90^ ^]^ The WHR curves of ST and RC specimens resemble those of conventional TRIP/TWIP Ti‐alloys,^[^ ^16^ ^]^ exhibiting maximum WHR values of ≈2.6 and ≈2.5 GPa respectively. Although the WHR of LTSTA specimen failed to reach those of ST and RC specimens, it was sufficient to match the progressively increasing flow stress, which is essential for maintaining high ductility.^[^ ^3^, ^29^ ^]^ RC and LTSTA specimens display identical 27.0% ε_T_ (true strain) uniform elongation based on Considère's criterion,^[^ ^91^ ^]^ evidencing without any ductility sacrifice (both uniform and fracture elongation) despite enhanced yield strength. To the best of our knowledge, the LTSTA specimen demonstrates the unprecedented yield strength‐ductility synergy (the product of yield strength and fracture elongation exceeds 38 GPa%) among all room‐temperature quasi‐static tensile tests of titanium alloys reported by far (Figure 2g).

### 2.3 Significant TRIP/TWIP Effects and Effective Work‐Hardening Contribution at High Flow Stress

**Figure 3:**
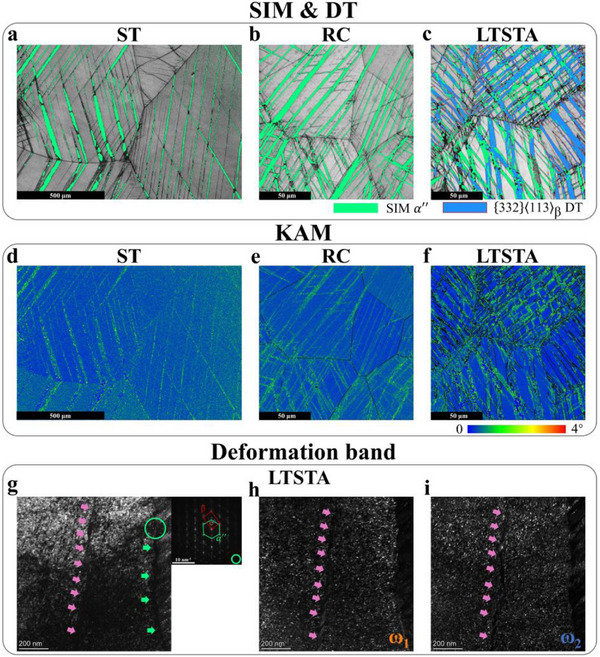
The deformation models of ST, RC, and LTSTA specimens at 0.02 ε_e_. a–c) phase maps superimposed with 332DT detection for ST, RC, and LTSTA specimens. d–f) Kernel Average Misorientation (KAM) maps for regions corresponding to a, b, c, respectively. g) bright field image of parallel‐aligned deformation band and SIM in the LTSTA specimen. Inset exhibits the SAED pattern captured from the region marked by the green circle. h,i) dark field images of two ω‐variants for region corresponding to g.

As shown in **Figure** **3**a,b, ST and RC specimens with low σ_y_ values exhibit TRIP‐only effect, which meets expectations of the low β phase stability design in Section 2.1. Although the RC specimen had a smaller grain size than the ST specimen, it triggered fine SIM that traversed β‐matrix grain boundaries (note that the scales of Figure 3a,b are not the same). This demonstrates the aforementioned complex influence of grain size on MT. Nonetheless, the mechanical performance of RC specimen, as compared to the coarse‐grained ST specimen, still indicates the effectiveness of fine‐grained strengthening still indicates the effectiveness of fine grain strengthening (Figure 2e). Reported ω‐enhanced TRIP/TWIP Ti alloys usually struggled to trigger intense TRIP/TWIP effects above 800 MPa σ_y_.^[^ ^15^, ^16^, ^92^ ^]^ However, owing to lean design of β phase stability and USFEs along β‐ω continuous slip, massive SIM and 332DT were observed in LTSTA specimen during early plastic stage, as shown in Figure 3c. Overlap of SIM and 332DT at multiple locations seemed to suggest the latter forms via mediation by the former, aiming to accommodate plastic strain under high flow stress. This will be discussed in the following Section.

Figure 3d–f present Kernel Average Misorientation (KAM) analysis for field of view corresponding to Figure 3a–c respectively. SIM acted as the primary deformation carrier in all three specimens. Comparatively, the LTSTA specimen experienced more severe deformation, which was characteristic of Lüders strain where localized plastic deformation exceeds macroscopic levels.^[^ ^90^ ^]^ Although SIM exhibits excellent synergistic deformation capability (Figure S9, Supporting Information), it is difficult to carry the ≈23.2% ε_e_ Lüders plateau only by the TRIP/TWIP effects.^[^ ^12^ ^]^ Consequently, slip behavior in LTSTA specimens was further analyzed. Typical ω‐induced localized dislocation activity is marked with pink arrows in Figure 3g–i, In particular, ω_1_ variants underwent β‐ω_1_ continuous slip shearing until dissolution below critical radius; ω_2_ variants reverted to β‐matrix with intensifying dislocation accumulation, forming ω_2_‐free dislocation channels.^[^ ^28^, ^42^ ^]^ Observed sparse deformation bands (DBs) indicate that slip did not take priority over TRIP/TWIP effects in LTSTA specimen, aligning with our design intention. Moreover, these DBs exhibited SIM‐parallel characteristics (Figure 3g; Figure S10, Supporting Information), signifying that micro local plasticity carried by slip cooperatively induced the yield drop and prolonged the Lüders plateau with SIM. Impoverished slip negated the potential for dislocation strengthening and the dynamic Hall‐Petch effect,^[^ ^16^ ^]^ while insufficiently hardened SIM resulted in negligible hetero‐deformation induced strengthening.^[^ ^1^, ^12^ ^]^ Consequently, premature necking within the Lüders plateau was predominantly delayed by local stress concentrations relaxation from dense TRIP/TWIP networks. During the uniform deformation stage post‐Lüders plateau, dislocation motion in both β‐matrix and SIM were anticipated to be adequately activated.^[^ ^93^ ^]^ The activated dislocation strengthening and dynamic Hall‐Petch effect collectively contributed to the ≈1.7 GPa WHR peak (Figure 2f).

### 2.4 Mechanism Underlying Dynamic Sequential Transformations in LTSTA Specimen

**Figure 4:**
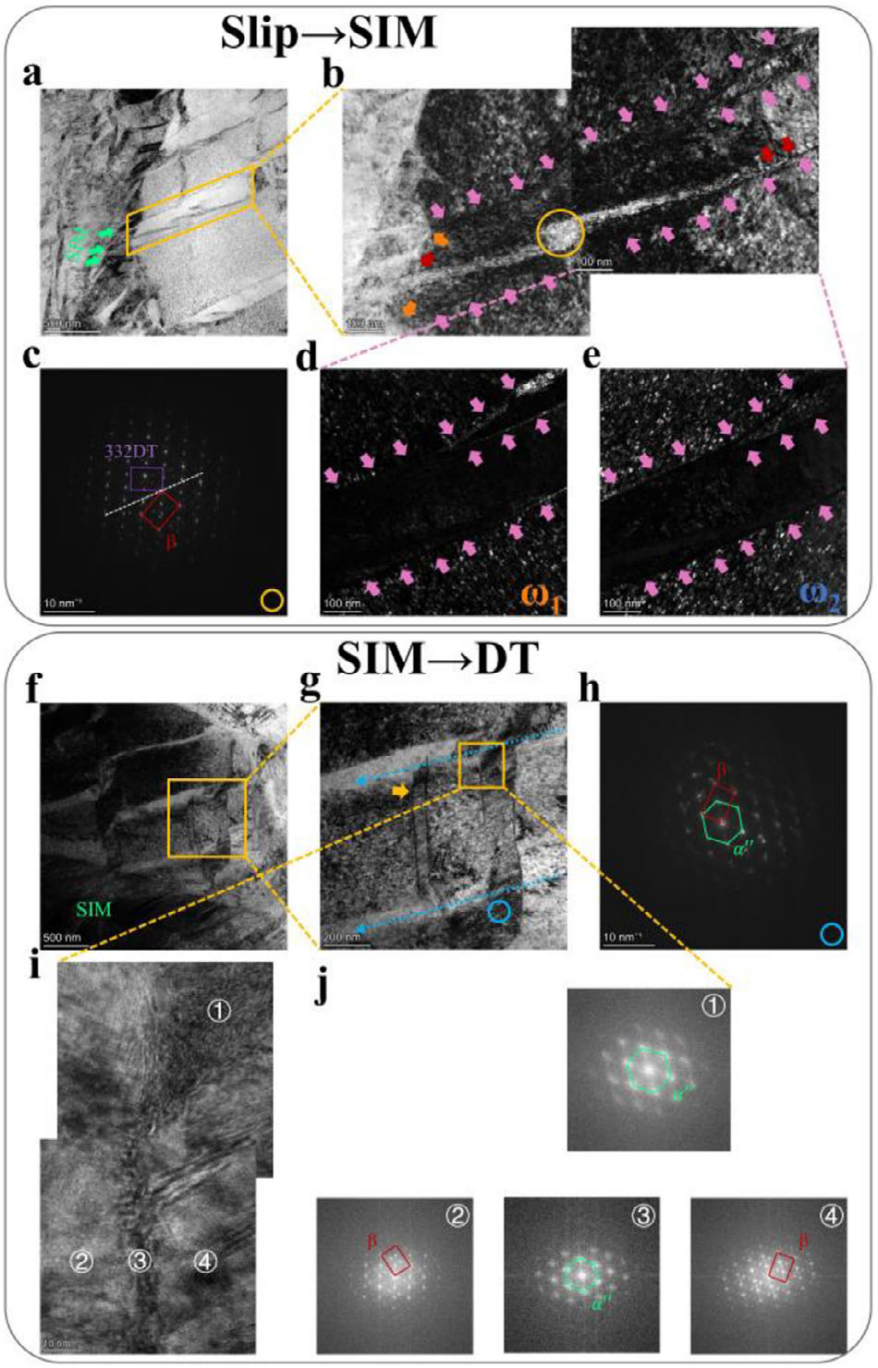
The sequential transformations in LTSTA specimen at 0.02 ε_e_. a,b) bright field image and localized magnification at the intersections of DBs and SIM. c) SAED pattern captured from the region marked by a yellow circle in b. d,e) dark field images of two ω‐variants for region corresponding to b. f,g) bright field image and localized magnification at the junction of SIM and 332DT. h) SAED pattern captured from the region marked by a blue circle in g. i) high resolution image of the region within the yellow box in g. j) fast Fourier transform images captured from the regions shown in i.

Stress‐induced martensite and strain‐induced martensite are different in concept.^[^ ^94^ ^]^ The emergence of the yield drop phenomenon in LTSTA specimen (Figure 2e) clearly indicates the deformation‐induced martensite is strain‐induced, with a prerequisite plastic deformation for its formation.^[^ ^94^ ^]^ MT in β phase is considered to comprise {112}<111> partial shear and {110}<110> shuffle steps.^[^ ^11^, ^31^ ^]^ In our design, ω‐confined β‐ω continuous slip along $\left(1\bar{1}2\right){\left[\bar{1}11\right]}_{\beta }$ and $\left(\bar{2}020\right){\left[0001\right]}_{\omega }$ and subsequent dislocation motion in ω‐free channels are expected to provide elevated local stress for MT‐essential shear. Therefore, the intersections between SIM and DBs were focused to provide rationality, as shown in **Figure** **4**a–e. Typical ω‐induced localized dislocation activities (pink arrows in Figure 4b,d,e) were observed to be trapped within SIM laths (orange arrows in Figure 4b). Furthermore, a banded structure identified as 332DT was captured (Figure 4b,c), with one end connected to the DBs and the other to SIM (red arrows in Figure 4b). Dislocation motion along {112}〈111〉_β_ slip system could not provide shear or shuffle contribution to directly formed 332DT.^[^ ^51^, ^95^ ^]^ Therefore, it is reasonable to speculate that the dislocation activity in ω‐free dislocation channel supplies enough local stress for lattice shear during SIM formation, subsequently inverting to 332DT here from size effect or higher stress.^[^ ^96^, ^97^ ^]^ The fully sequential transformation that mitigates local stress concentration was systematically summarized in the schematic diagram, as shown in Figure S11 (Supporting Information).

Three positional relationships of SIM‐mediated 332DT formation were observed, jointly causing a complex overlap in the electron backscatter diffraction (EBSD) analysis (Figure 3c). Figure 4b and Figure S12 (Supporting Information) show that 332DT formed epitaxially and internally with SIM, respectively. Moreover, sandwich structures featuring SIM at both ends and 332DT in the middle section were observed (Figure 4f–j). The 332DT interface exhibits an α′′ structure and is connected to SIM at the top end, which was considered an incompletely transformed product, rather than the relaxed 332DT interface structure^[^ ^51^ ^]^ in low β‐stability titanium alloys. A TEM analysis of the structure marked by yellow arrow in Figure 4g further supported this conclusion, that is, both interfaces of 332DT connect with the top SIM and exhibit α′′ structures with consistent orientation (Figure S13, Supporting Information). Sandwich structures emerged to relieve local stress concentration induced by shear deformations perpendicular to SIM (blue dotted arrows in Figure 4g) under high flow stress. Similarly, overlap between SIM and 332DT predominantly occurred at grain boundaries of β‐matrix and intersections of TRIP/TWIP networks (Figure 3c). Although the dynamic sequential transformation from SIM to 332DT has been previously reported,^[^ ^95^, ^96^ ^]^ its accommodation capability for high flow stress in this work is remarkably significant. Therefore, sustainably tuning the balance between slip and TRIP/TWIP effects by manipulating β phase stability and β‐ω continuous slip barriers is a promising method to further overcome the yield strength‐ductility trade‐off in titanium alloys.

## 3 Conclusion

Comprehensive and rigorous theoretical calculations were performed in this study to screen potential compositions for coupling ω‐nanoprecipitates with TRIP/TWIP effects in titanium alloys. Diverse interactions between sparse DBs and SIM, and between SIM and 332DT, were captured to reveal ω‐confined specific shear promoting MT and the MT→332DT sequential transformation. During the ≈23.2% ε_e_ Lüders plateau, the former transformation accommodates primary plastic deformation, while the latter transformation postpones necking by relieving stress concentration. During the subsequent uniform deformation, ≈1.7 GPa WHR peak indicates activation of dislocation strengthening and dynamic Hall‐Petch effect. This enables the alloy to achieve > 45% ε_f_ at > 830 MPa σ_y_, with their synergy (product exceeds 38 GPa%) surpassing that of all reported titanium alloys. Our methodology and design strategies are expected to be extendable to other TRIP/TWIP‐type alloy systems for breaking the low yield strength cruse without sacrificing ductility.

## 4 Experimental Section

### Theoretical Calculation Methods

All density functional theory (DFT) calculations were conducted using the Vienna Ab Initio Simulation Package (VASP).^[^ ^98^ ^]^ The exchange‐correlation functional was described by generalized gradient approximation of the Perdew‐Burke‐Ernzerhof form (GGA‐PBE) with the projector augmented wave (PAW) method.^[^ ^99^, ^100^ ^]^ Elements pseudopotentials were chosen following VASP recommendation. Γ‐centered k‐point mesh was used to implement Brillouin zone sampling, with mesh size smaller than 2π*0.03 (1/Å). The plane‐wave cutoff energy, energy and force convergence threshold were set as 500 eV, 1×10^−6 ^eV/atom, and 2×10^−2^ eV/Å (3×10^−2^ eV/Å for elastic calculations^[^ ^48^ ^]^), respectively. To account for atomic distribution characteristics, 4[100]×2[$0\bar{1}1$]×3[011] and 2[$\bar{1}10$]×2[111]×2[$11\bar{2}$] BCC supercells were first constructed using the special quasiordered structure (SQoS) method,^[^ ^101^, ^102^ ^]^ followed by subsequent generation of α′′ and ω structures through crystal structure transformations.^[^ ^40^, ^103^ ^]^ Coordination‐neighbor (C‐n) relaxation was performed to optimize BCC configurations,^[^ ^104^, ^105^ ^]^ considering the mechanical/dynamic instability of BCC‐Ti at 0 K.^[^ ^38^ ^]^ For optimization of α′′ and ω structures, volume, shape and atomic positions were full relaxed. In stacking fault energies calculations, the model shape and volume were fixed, with atomic relaxation permitted exclusively perpendicular to the slip plane. Elastic properties were obtained through the stress–strain method, with both execution and post‐processing conducted using VASPKIT.^[^ ^106^ ^]^ Visualization was performed using VESTA and OVITO packages.^[^ ^107^, ^108^ ^]^

The Alloy Theoretic Automated Toolkit (ATAT) package^[^ ^109^ ^]^ was used to perform Cluster expansion (CE), Monte Carlo (MC) simulations and SQoS constructions. Configurations were optimized using structural relaxation settings identical to those described above to build the training set. After excluding configurations exhibiting excessive deviation from BCC structure via checkrelax function in ATAT, 1015 structures were employed to construct the configurational energy expression with leave‐one‐out cross‐validation score as low as 20 meV atom^−1^. The constructed configurational energy expression was utilized to track energy variations in canonical ensemble MC simulations, which were executed using the memc2 code in ATAT. 20 × 20 × 20 BCC disorder supercells (16 000 atoms) were used as initial configurations to cool from 3273 to 73 K. Repeated simulations of identical configurations were performed for approximating statistical average. At each temperature, thermodynamic equilibrium was determined through an energy convergence threshold of 1 × 10^−5^ eV/atom.^[^ ^110^ ^]^ The average correlations associated with each cluster at 1173 K in MC simulations were designated as the objective function of SQoS, which corresponds to the experimental homogenization annealing temperature.

Ab Initio Molecular Dynamics (AIMD) simulations based on VASP were performed at 300 K on 2 × 1 × 1 built ω models (192 atoms) and 1 × 2 × 1 built α′′ models (192 atoms) with Γ‐point mesh. Computational parameters were maintained identical to preceding DFT calculations. To further restrict crystal structure evolution exclusively within independent ω→β and α′′→β pathways, NVT (Nosé–Hoover thermostat) and NPT (Langevin thermostat) ensembles were employed for the former and the latter respectively, due to the ability of the NVT ensemble to suppress potential lattice strain associated with MT.^[^ ^39^ ^]^ All AIMD simulations ran 40 ps with a time step of 2 fs. The average lattice parameters and atomic coordinates of the last 10 ps were regarded as thermodynamic average configurations. For effective diffusion coefficients based on vacancy diffusion mechanisms,^[^ ^111^ ^]^ one Ti atom was intentionally removed to initiate AIMD simulations, with the mean squared displacement during the last 35 ps used to determine diffusion coefficients.

### Materials and Processing

High‐purity elementary substances (99.98 wt.% Ti, 99.95 wt.% Mo, 99.9 wt.% Cr, 99.9 wt.% Zr) were arc‐melted five times under argon atmosphere to produce 65 × 45 × 15 mm^3^ ingots. Rectangular specimens (23 × 9 × 14 mm^3^) were sectioned from the ingots using electrical discharge machining. As shown in Figure 1c1, the rectangular specimens subjected to homogenization treatment at 1173 K for 1 h followed by water quenching, which corresponds to the solution‐treated (ST) specimen. Subsequent thickness reduction from 14 to 2.5 mm was achieved via cold rolling procedures. Recrystallization treatment was implemented at 1123 K for 10 min with subsequent water quenching, which corresponds to the RC specimen. Finally, low‐temperature and short‐time aging (LTSTA) were performed at 483 K for 60 s in the oil bath and then quenched in water, which corresponds to the LTSTA specimen.

### Experimental Methods

For the room‐temperature quasi‐static uniaxial tensile test, the samples with gauge dimensions of 2.5 × 3 × 15 mm^3^ were cut from the metal plates after the above treatment. Tensile tests were performed in Instron5982 with the contacted extensometer at a strain rate of 1 × 10^−3^ s^−1^. Three specimens were tested to verify the reproducibility. The LVE‐5M camera was used to perform the digital image correlation (DIC)‐based in situ tensile test. The Ncorr software^[^ ^112^ ^]^ was applied to analyze the recorded images. For electron backscatter diffraction (EBSD) and transmission electron microscope (TEM) observations, the specimens were prepared by electrolytic polishing and twin‐jet electropolishing, using a solution of 5% perchloric acid, 35% n‐butanol and 60% methanol at −30 °C. A SEM type CIQTEK SEM5000 equipped with an EBSD type Oxford Symmetry2 (20 kV), and a TEM type Thermofisher Talos F200X (200 kV) were used for microstructure investigations.

## Conflict of Interest

The authors declare no conflict of interest.

## Supporting information



Supporting Information

## Data Availability

The data that support the findings of this study are available from the corresponding author upon reasonable request.
