# Supplementary material for: TRIP/TWIP Networks Promoted via Multifunctional Nanoprecipitates‐confined Specified Shear for Achieving Strong‐yet‐Ductile Titanium Alloys
Source: Adv Sci (Weinh). 2025 Sep 22;12(44):e11834. doi: 10.1002/advs.202511834 (PMC12667494; doi:10.1002/advs.202511834)
Supplement: Supplementary file 1 — Supporting Information [file ADVS-12-e11834-s001.pdf]

**Supplementary Information for:**

# TRIP/TWIP Networks Promoted via Multifunctional Nanoprecipitates-confined Specified Shear for Achieving Strong-yet-ductile Titanium Alloys

*Xiaofu Zhang, Shu Wang*, Ruirun Chen*, Minghao Hua, Weipeng Xu, Hongwei Wang, Shuo Yin*

## Affiliations

X. Zhang, S. Wang, R. Chen, W. Xu, H. Wang

National Key Laboratory for Precision Hot Processing of Metals

School of Materials Science and Engineering

Harbin Institute of Technology

Harbin 150001, PR China

E-mail: wangshu@hit.edu.cn; ruirunchen@hit.edu.cn

M. Hua

School of Energy and Power Engineering

Shandong University

Jinan 250061, PR China

S. Yin

Department of Mechanical, Manufacturing and Biomedical Engineering

Trinity College Dublin, The University of Dublin

Dublin 2, Ireland

## Note 1 Determination of LTSTA process parameters

Diffusion behaviors in large system at second-level durations cannot be captured by current atomic-scale simulations. On the contrary, setting the aging time consistent with reported literature[1] enables the design of LTSTA process parameters by leveraging our phase stability calculation results. First, the objective of LTSTA treatment has been established based on related literatures:[1–3] avoid ω phase growth in size, number density, and volume fraction (high ω-embrittlement risk), while permitting only short-range solute diffusion between ω phase and matrix. Then, we clarified aging temperature selection as follows. Ideally, β-matrix and athermal ω phase share identical composition (diffusionless transformation), without solute concentration gradients. Solute diffusion is thus driven primarily by chemical driving force and thermal activation. That is to say, compositions with lower chemical driving forces require higher temperatures to activate diffusion. Chemical driving force could be indirectly reflected through our calculations on β phase stability during ω-transition (Figure 1b). Reported experimental studies showed initial temperatures during heating of $\omega_{iso}$ transformation ($T_{\omega_{iso}}$) for Ti-12Mo[4] and Ti-15Mo[5] alloys are 451 K and 498 K respectively, only 47 K temperature window. As shown in Figure 1b, the β phase stability of designed alloy lies between Ti-12Mo and Ti-15Mo alloys, closer to the former. Thus, its $T_{\omega_{iso}}$ is expected to be slightly higher than Ti-12Mo alloy.

Additionally, the diffusion rate of the compositions should be considered to achieve short-range solute diffusion within a limited time. Based on reported calculation methods[6], we calculated vacancy diffusion coefficient for β phase with different compositions. As shown in Supplementary Figure 2, diffusion coefficient of designed composition also resides between Ti-12Mo and Ti-15Mo alloys. Therefore, at identical aging times, setting the aging temperature of our alloy slightly higher than the optimal aging temperature of Ti-12Mo alloy (473 K)[1] could compensate for both chemical driving force and diffusion rate.

## Note 2 The potential implications of LTSTA process parameters on thicker sections

To reveal the potential implications of LTSTA process on thicker sections, we performed finite element analysis via *Comsol Multiphysics* software to simulated LTSTA process (heating and water quenching) based on the solid heat transfer model, as shown in Supplementary Figure 3. The alloy's thermal conductivity was set to 8 W/(m·K) (298 K) - 12 W/(m·K) (483 K), consistent with common titanium alloys. The time difference between the specimen surface and core reaching target temperatures (i.e., 483 K for heating and room temperature for water quenching) served as the criterion to determine the maximum thickness. As shown in Supplementary Figure 3b and c, when the time difference threshold was set to 3 s (representing a 5% error relative to the 60 s of LTSTA process), the maximum allowable thicknesses for heating and water quenching were 7 mm and 6 mm, respectively. Consequently, within the permissible 6 mm thickness, we consider the LTSTA process holds wide application potential in engineering field.

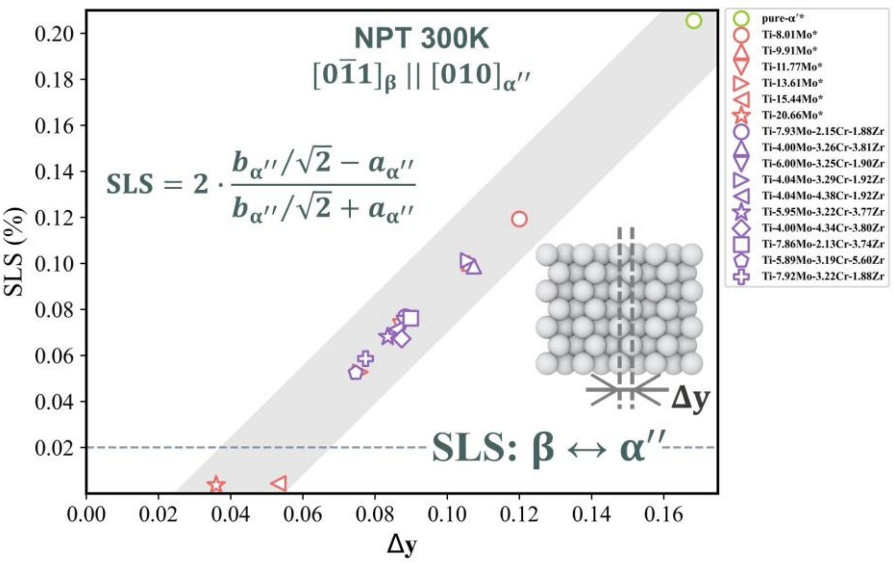


**Supplementary Figure 1** The spontaneous lattice strain and average atomic shuffle (Δy) of equilibrium structures with $\alpha''$ phase as initial configuration for different compositions. Asterisked data derives from [our unpublished results].

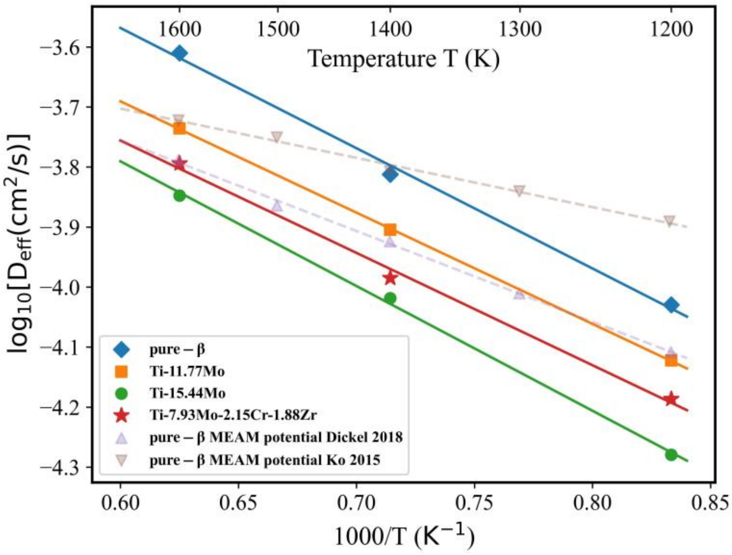


**Supplementary Figure 2** The effective diffusivity as a function of temperature for different compositions. Comparative data represented by the triangle symbols were extracted from the reported literature.[7]

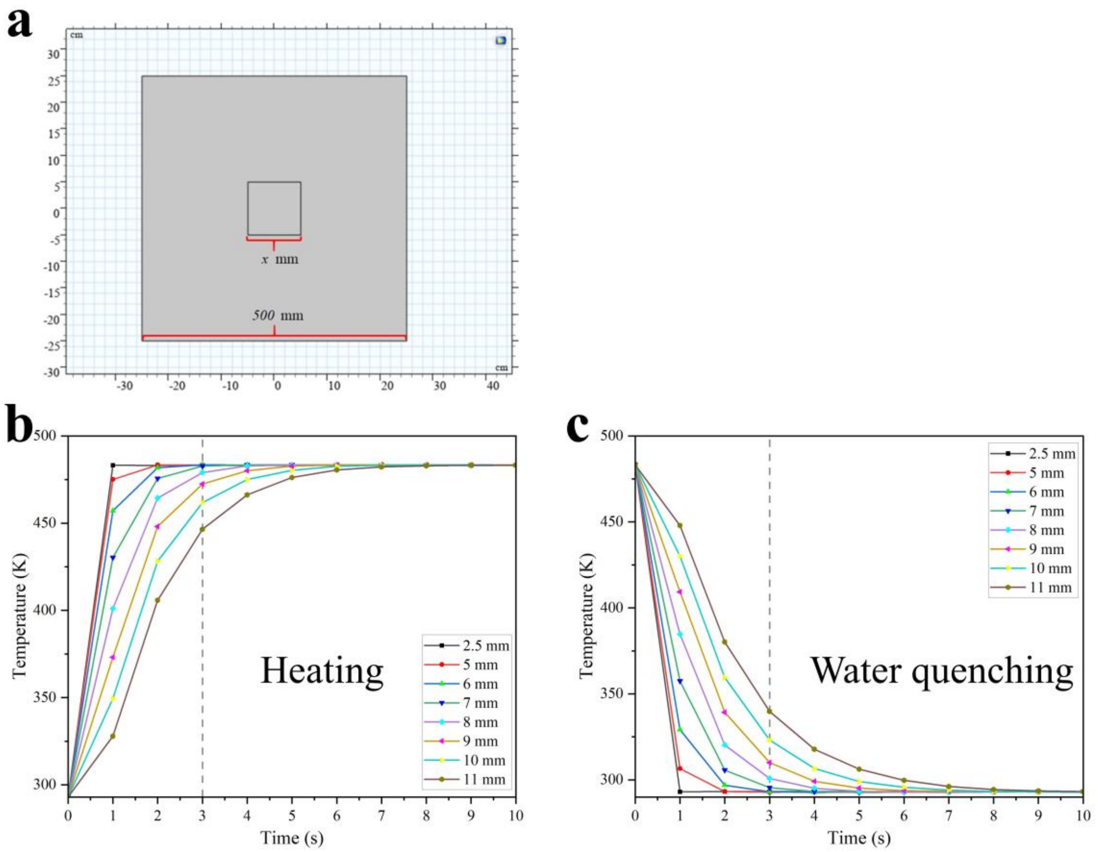


**Supplementary Figure 3 a**, finite element model and mesh. **b**, **c**, under the condition of heating and water quenching, the effect of wall thickness on the time difference between the core and the surface to reach the target temperature.

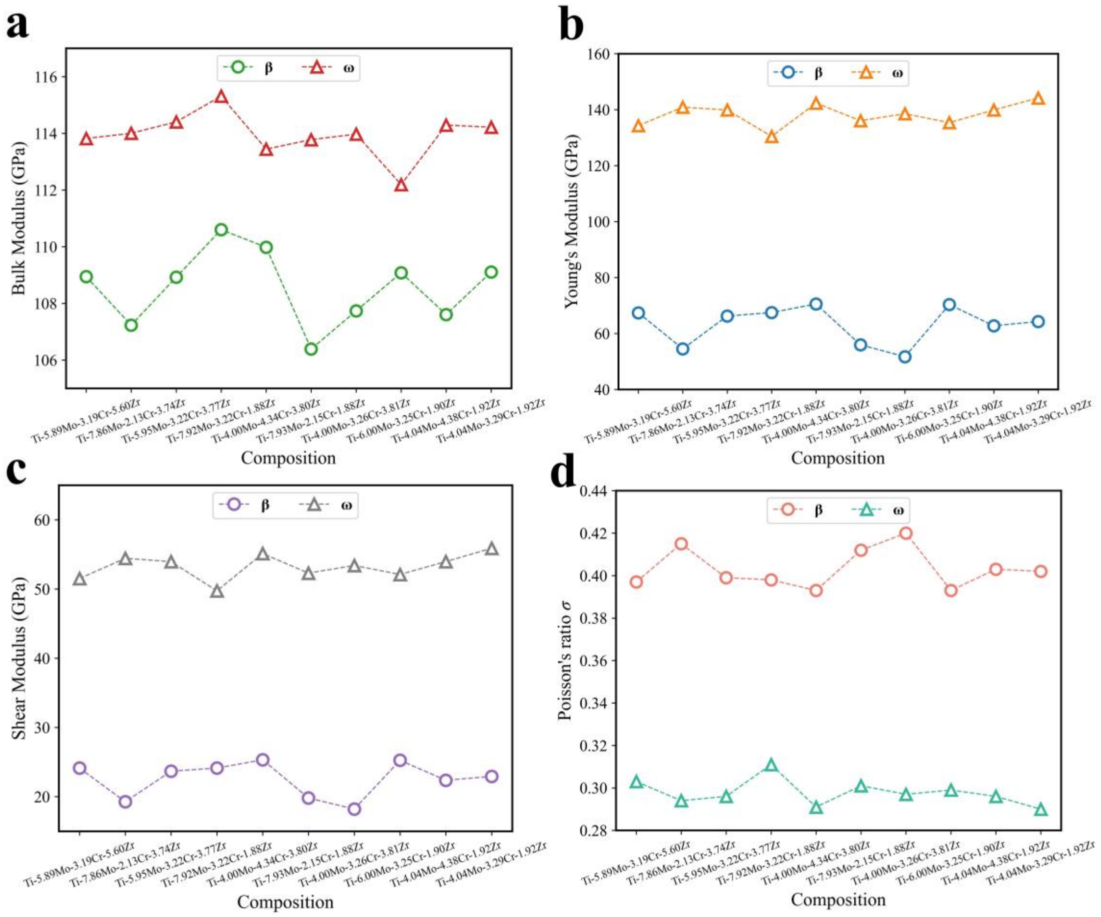


**Supplementary Figure 4** The elastic properties of β and ω phase in Ti-Mo-Cr-Zr alloys. **a**, bulk modulus. **b**, Young's modulus. **c**, shear modulus. **d**, poisson's ratio.

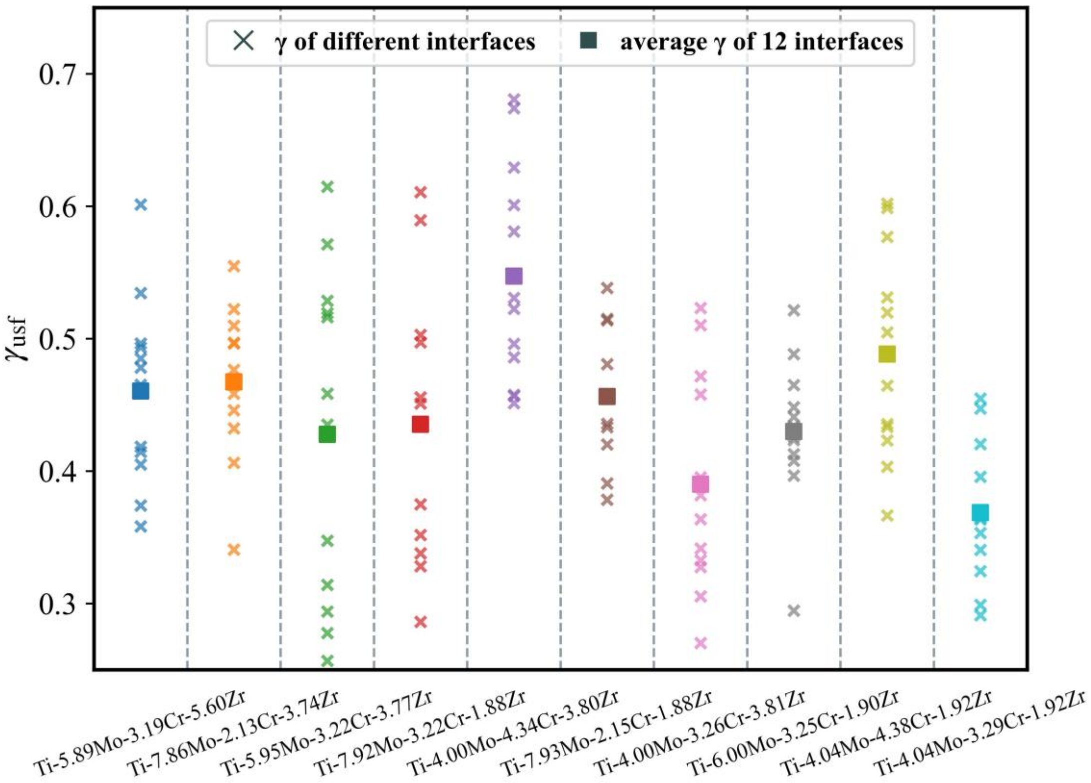


**Supplementary Figure 5** The unstable stacking fault energies (USFEs) of different planes and the average USFEs of all planes in β phase for Ti-Mo-Cr-Zr alloys.

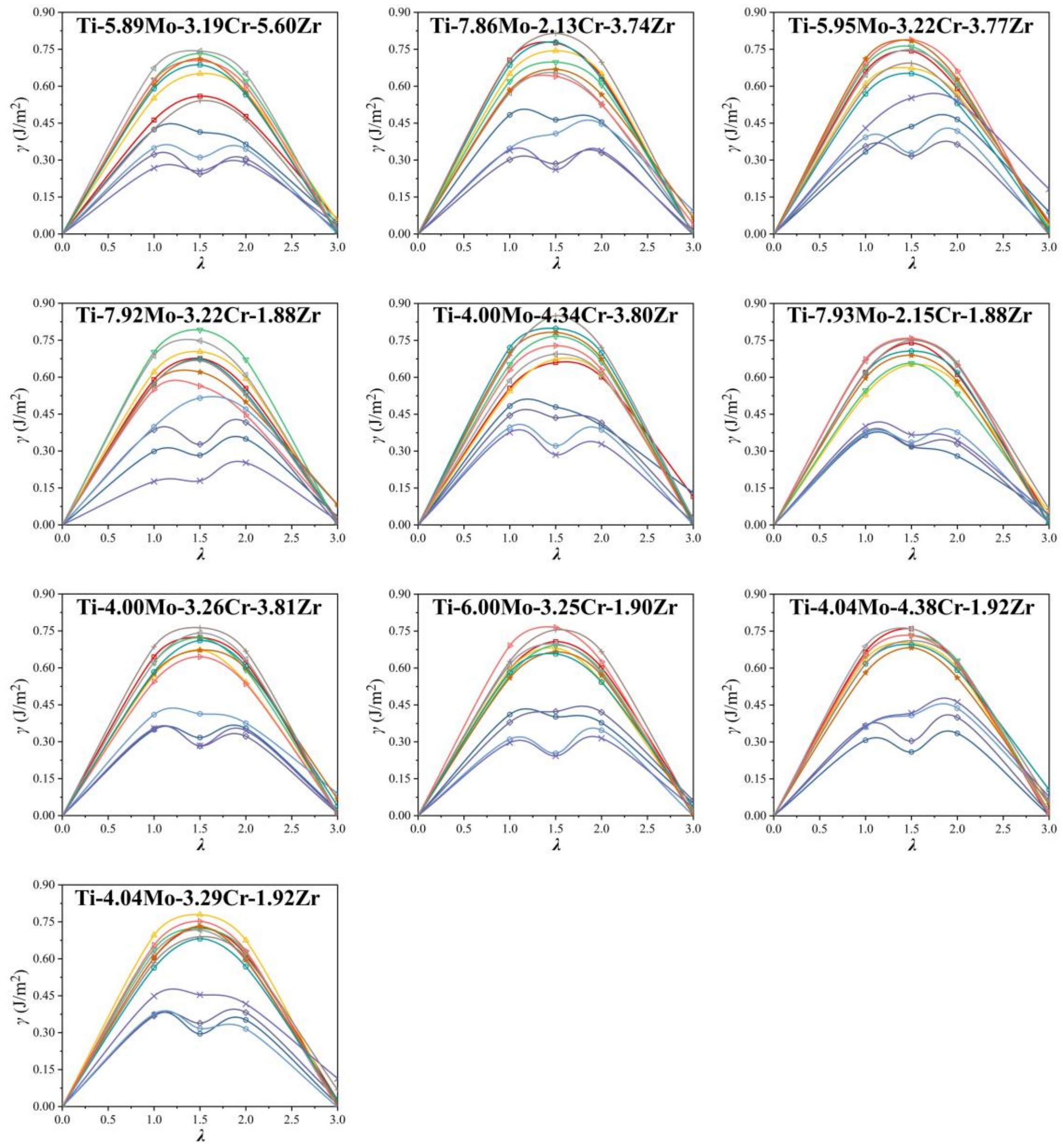


**Supplementary Figure 6** the stacking fault energy curves of all planes of ω phase in Ti-Mo-Cr-Zr alloys.

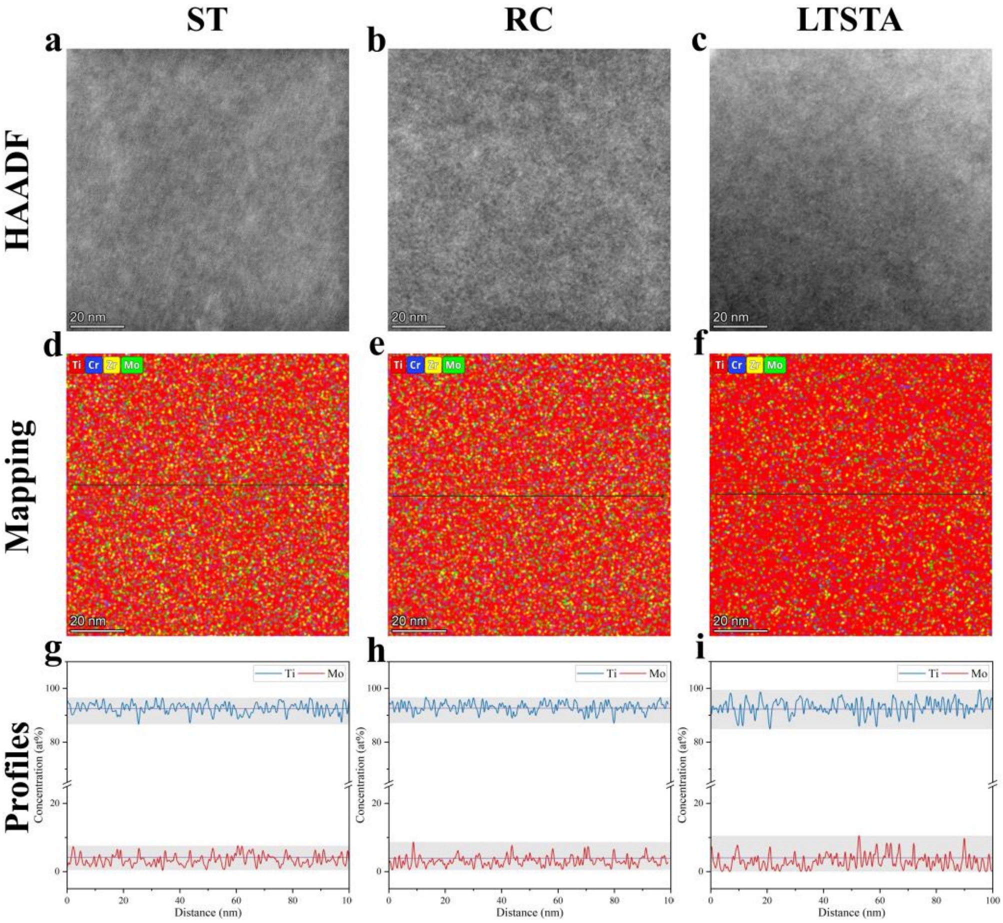


**Supplementary Figure 7 a-c**, HAADF images. **d-f**, elements mapping. **g-i**, element concentration profiles along lines in **d-f**.

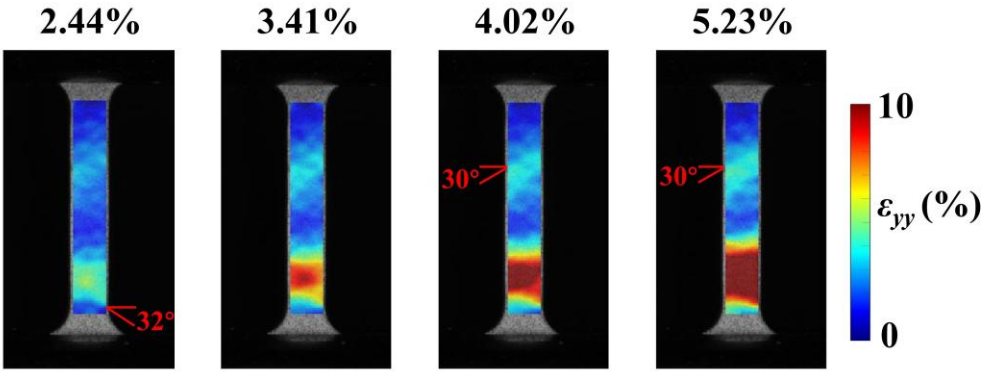


**Supplementary Figure 8** DIC analysis of LTSTA specimen.

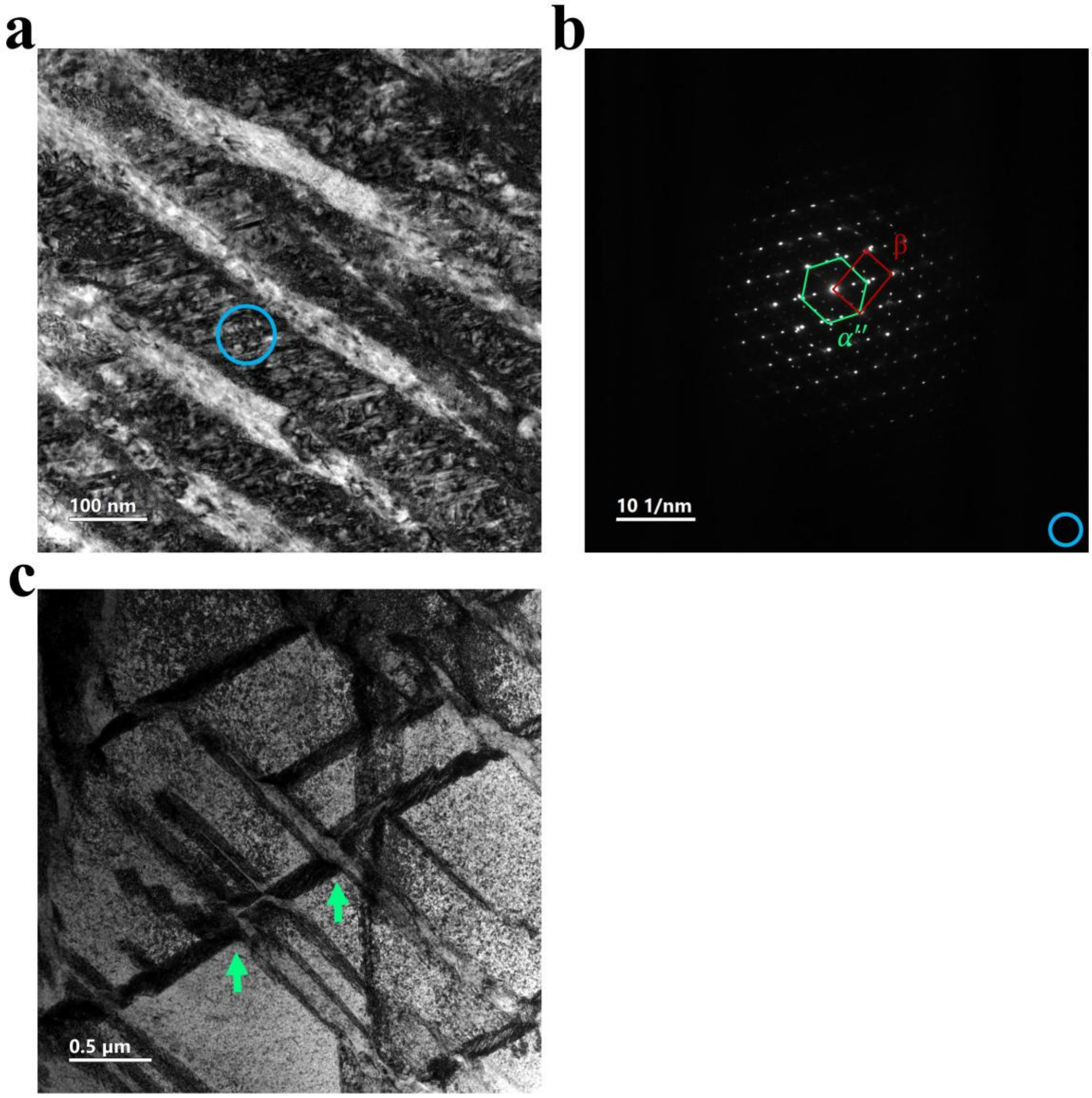


**Supplementary Figure 9 a**, **c**, bright images. **b**, SAED pattern captured from blue circle region in **a**.

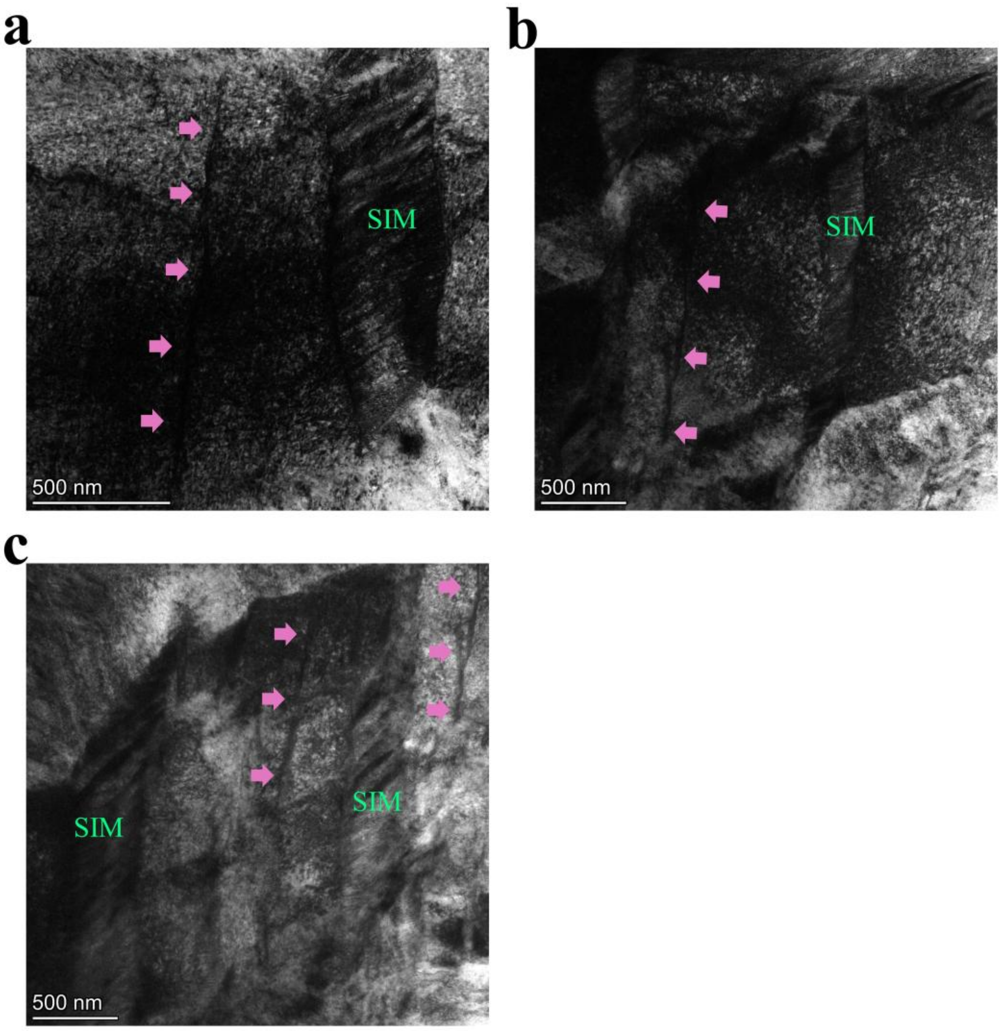


**Supplementary Figure 10** Bright field images of parallel relations between deformation bands and SIM.

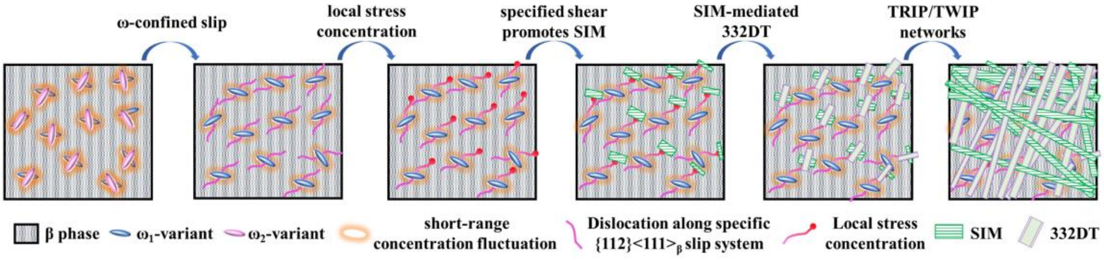


**Supplementary Figure 11** Schematic diagram of fully sequential transformation.

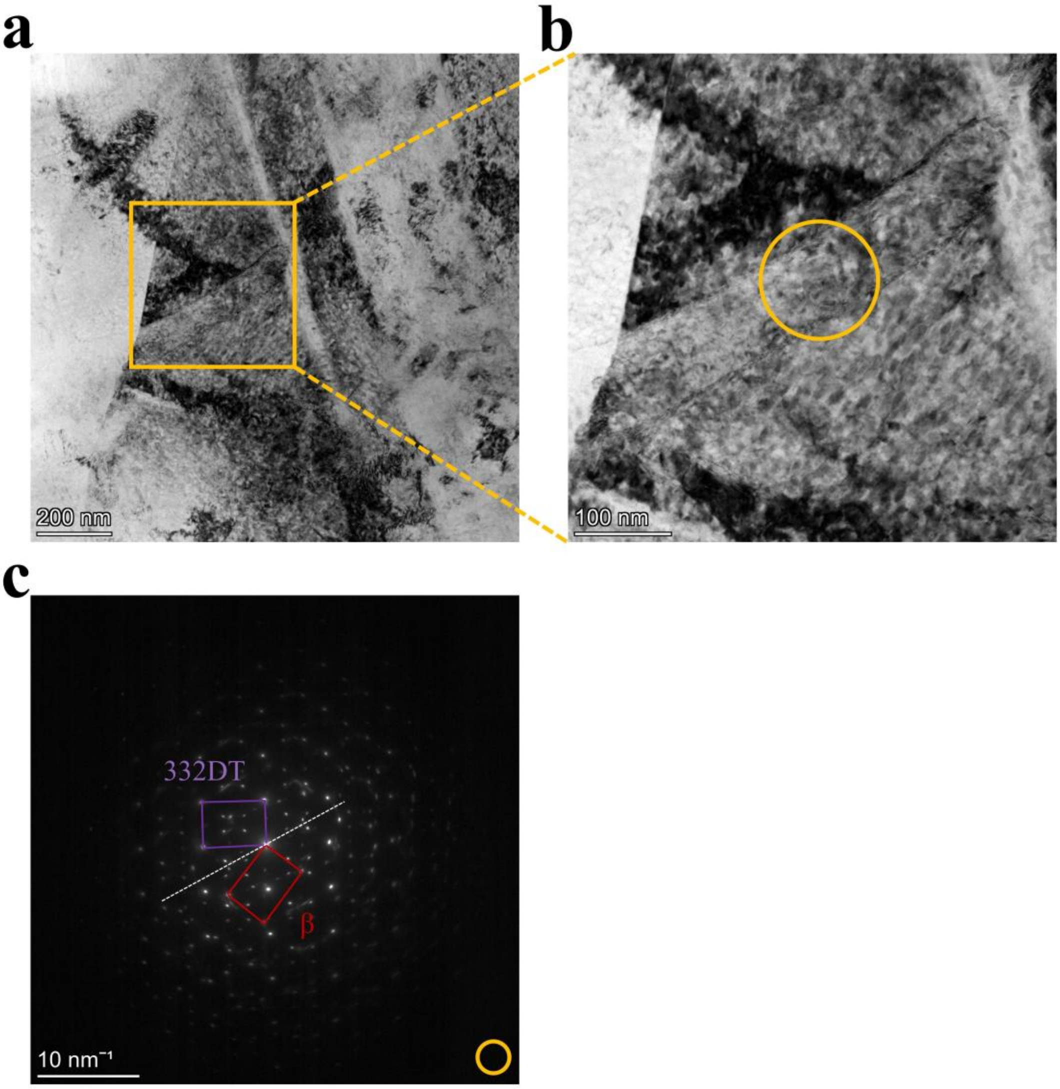


**Supplementary Figure 12 a**, **b**, bright field image and localized magnification of 332DT. **c**, SAED pattern captured from yellow circle region in **b**.

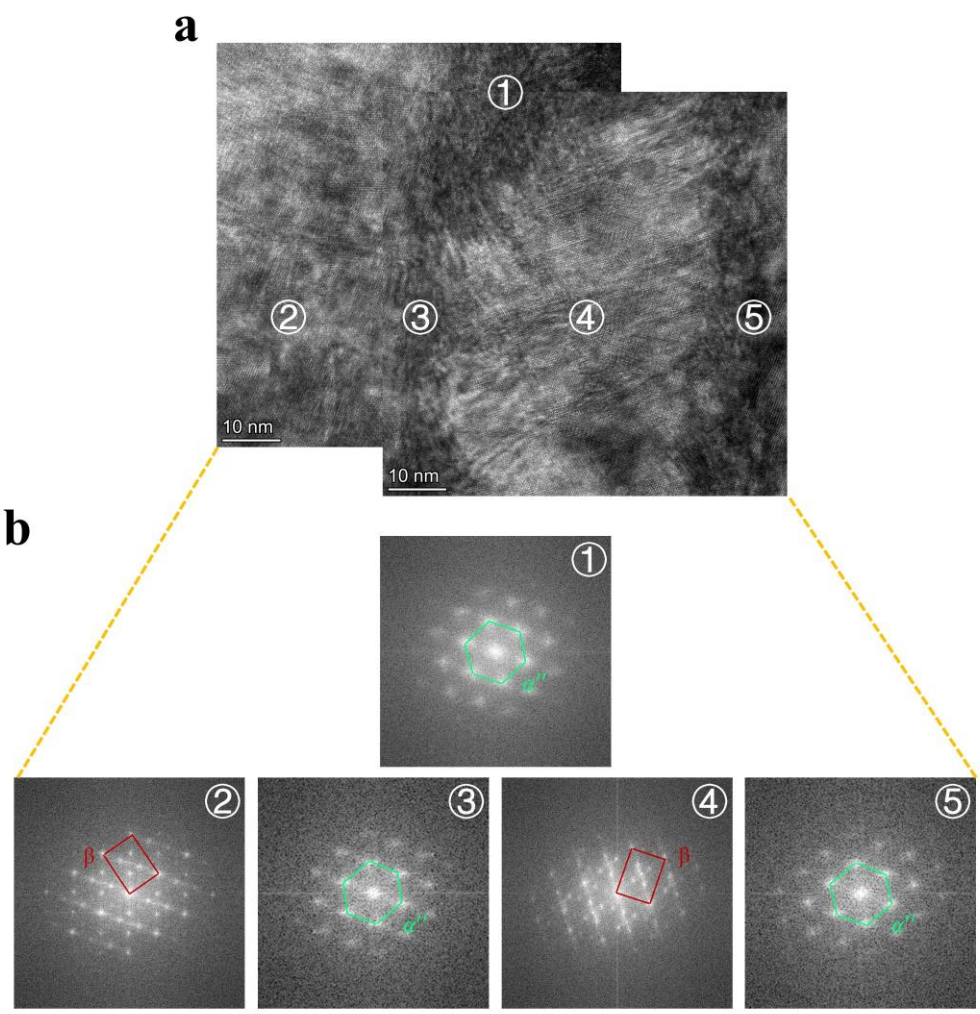


**Supplementary Figure 13 a**, high resolution image of the region indicated by the yellow arrow in **Figure 4g**. **b**, FFT images captured from the regions shown in **a**.

**Supplementary Table 1** USFEs and Shear modulus of Ti-39.58 at.% Mo β phase

| Ti-39.58Mo | P1 | P2 | P3 | P4 | P5 | P6 | P7 | P8 | P9 | P10 | P11 | P12 | AVG |
|---|---|---|---|---|---|---|---|---|---|---|---|---|---|
| $\gamma_{\beta}^{usf}$ (J/m2) | 0.73 | 0.97 | 0.89 | 1.04 | 1.03 | 1.01 | 0.99 | 0.89 | 0.90 | 0.88 | 0.77 | 0.81 | 0.91 |
| $G_{\beta}$ (GPa) | | | | | | | 48.40 | | | | | | |

**Supplementary Table 2** Quantitative statistics of microstructure characteristics for ω phase in three states specimens based on TEM dark field images

| Alloy state | Size $d_0$ (nm) | Volumic numerical density $\bar{N}_{\omega}$ ($m^{-3}$) | Volume fraction $f$ (%) |
|---|---|---|---|
| ST | 5.54 | $8.67\times10^{23}$ | 7.72 |
| RC | 5.86 | $7.27\times10^{23}$ | 7.66 |
| LTSTA | 5.65 | $7.98\times10^{23}$ | 7.52 |

## References

[1] B. Qian, S. A. Mantri, S. Dasari, J. Zhang, L. Lilensten, F. Sun, P. Vermaut, R. Banerjee, F. Prima, *Acta Materialia* **2023**, *245*, 118619.
[2] F. Sun, J. Y. Zhang, P. Vermaut, D. Choudhuri, T. Alam, S. A. Mantri, P. Svec, T. Gloriant, P. J. Jacques, R. Banerjee, F. Prima, *Materials Research Letters* **2017**, *5*, 547.
[3] M. J. Lai, T. Li, F. K. Yan, J. S. Li, D. Raabe, *Scripta Materialia* **2021**, *193*, 38.
[4] F. Sun, D. Laillé, T. Gloriant, *J Therm Anal Calorim* **2010**, *101*, 81.
[5] P. Zháňal, P. Harcuba, M. Hájek, B. Smola, J. Stráský, J. Šmilauerová, J. Veselý, M. Janeček, *J Mater Sci* **2018**, *53*, 837.
[6] Z. Su, J. Ding, M. Song, L. Jiang, T. Shi, Z. Li, S. Wang, F. Gao, D. Yun, E. Ma, C. Lu, *Acta Materialia* **2023**, *245*, 118662.
[7] G. Smirnov, *Phys. Rev. B* **2020**, *102*, 184110.
